# C1QC is a prognostic biomarker with immune-related value in kidney renal clear cell carcinoma

**DOI:** 10.3389/fgene.2023.1109991

**Published:** 2023-03-13

**Authors:** Wentao Yao, Hanyuan Liu, Feng Xu, Zhenyu Cai, Lijing Hang, Mingya Lu, Yuan Zhao, Chendi Yang, Yang Zong

**Affiliations:** ^1^ Department of Urology, Suzhou TCM Hospital Affiliated to Nanjing University of Chinese Medicine, Suzhou, China; ^2^ Department of General Surgery, Nanjing First Hospital, Nanjing Medical University, Nanjing, China; ^3^ Laboratory of Clinical Pharmacy of Traditional Chinese Medicine, Suzhou TCM Hospital Affiliated to Nanjing University of Chinese Medicine, Suzhou, China

**Keywords:** C1QC, kidney renal clear cell carcinoma, prognosis, immune infiltration, tumor microenvironment

## Abstract

**Background:** Kidney renal clear cell carcinoma (KIRC) is a representative histologic subtype of renal cell carcinoma (RCC). RCC exhibits a strong immunogenicity with a prominent dysfunctional immune infiltration. Complement C1q C chain (C1QC) is a polypeptide in serum complement system and is involved in tumorigenesis and the modulation of tumor microenvironment (TME). However, researches have not explored the effect of C1QC expression on prognosis and tumor immunity of KIRC.

**Methods:** The difference in a wide variety of tumor tissues and normal tissues in terms of the C1QC expression was detected using TIMER and TCGA portal databases, and further validation of protein expression of C1QC was conducted *via* Human Protein Atlas. Then, the associations of C1QC expression with clinicopathological data and other genes were studied with the use of UALCAN database. Subsequently, the association of C1QC expression with prognosis was predicted by searching the Kaplan-Meier plotter database. A protein-protein interaction (PPI) network with the Metascape database was built using STRING software, such that the mechanism underlying the C1QC function can be studied in depth. The TISCH database assisted in the evaluation of C1QC expression in different cell types in KIRC at the single-cell level. Moreover, the association of C1QC and the infiltration level of tumor immune cell was assessed using TIMER platform. The TISIDB website was selected to deeply investigate the Spearman correlation between C1QC and immune-modulator expression. Lastly, how C1QC affected the cell proliferation, migration, and invasion *in vitro* was assessed using knockdown strategies.

**Results:** KIRC tissues had notably upregulated C1QC level in comparison with adjacent normal tissues, with showed a positive relevance to clinicopathological features including tumor stage, grade, and nodal metastasis, and a negative relevance to clinical prognosis in KIRC. C1QC knockdown inhibited KIRC cell proliferation, migration, and invasion, as indicated by the results of the *in vitro* experiment. Furthermore, functional and pathway enrichment analysis demonstrated that C1QC was involved in immune system-related biological processes. According to single-cell RNA analysis, C1QC exhibited a specific upregulation in macrophages cluster. Additionally, there was an obvious association of C1QC and a wide variety of tumor-infiltrating immune cells in KIRC. Also, high C1QC expression presented inconsistent prognosis in different enriched immune cells subgroups in KIRC. Immune factors might contribute to C1QC function in KIRC.

**Conclusion:** C1QC is qualified to predict KIRC prognosis and immune infiltration biologically. Targeting C1QC may bring new hope for the treatment of KIRC.

## Introduction

Renal cell carcinoma (RCC) comprises 4% of all malignancies and is the eighth most common cancer in the United Sates (Siegel et al., 2022). In China, a total of 75,800 newly diagnosed RCC cases and 26,900 RCC-related deaths are estimated to occur in 2016 (Zheng et al., 2022). As the major subtype of RCC, kidney renal clear cell carcinoma (KIRC) accounts for 75%–80% of the RCC diagnoses (Moch et al., 2022). However, nearly 25% patients developed metastasis at initial diagnosis of KIRC and, even worse, median survival for the above patients only reaches nearly 13 months (Cohen and McGovern, 2005). The past decades have witnessed revolutions in both KIRC understanding and treatment. To date, KIRC is considered to exhibit a strong immunogenicity, and we are in the era of immune checkpoint inhibitors. However, a considerable number of patients still have poor immunotherapy response and develop resistance to immunotherapy after being treated for a long term (Motzer et al., 2022). Hence, it is necessary to elucidate the immunophenotype of tumor-immune interactions and discover novel therapeutic targets related to immune in KIRC.

Tumor microenvironment (TME) comprises immune cells, extracellular matrix, immunomodulators, stromal cells, and tumor cells. Immune cells and molecules in TME jointly facilitate tumor immune escape, tumor growth, and metastasis, though they cannot enter tumor tissue (Peranzoni et al., 2018). Recent research has suggested that the complement system has an effect of immunoregulation associated with tumorigenesis in TME (Afshar-Kharghan, 2017). Complement C1q C chain (C1QC) is a polypeptide involved in the production of C1 which is the first component of the serum complement system. Functionally, C1QC can regulate a wide variety of fundamental pathological and physiological processes including occurrence and development of cancer, removal of immune complexes, inflammation of body, and apoptosis of cells (Son et al., 2015). C1QC is overexpressed among different TMEs (Ain et al., 2021), and its potential cancer-promoting effect has been reported in several studies. C1QC was reported to be elevated in soft tissue sarcoma and associated with worse prognosis (Zhang et al., 2020). Qi Yang et al. demonstrated that high expression of C1QC in female-derived tumor-associated macrophages was related to poor prognoses in non-small cell lung cancer (Yang et al., 2021). Notably, existing research has also suggested that inhibition of C1QC expression of tumor-associated macrophages can suppress the differentiation from M1 to M2 macrophages and inhibit the growth of digestive system cancer cells (Hui et al., 2022). However, C1QC overexpression reported a better skin cutaneous melanoma prognosis (Yang et al., 2022), not consistent with the promotive role of C1QC in cancer progression as mentioned in existing research. Thus, C1QC has multifaceted functions in TME.

However, there are few studies on the specific role of C1QC in KIRC, and its related prognosis and possible immune mechanisms are still ambiguous. In the present study, bioinformatics analysis and specific cell experiments were employed to explore function of C1QC in KIRC. The clinical relevance, the potential molecular mechanisms, and the association of C1QC with TME were studied by examining C1QC expression in KIRC. The findings of this study provide more insights into the effect of C1QC on KIRC and the possible regulatory function of C1QC over immune cell infiltration (such regulatory function partially impacted KIRC prognosis).

## Materials and methods

### C1QC expression level analysis and clinicopathological analysis

Table 1 all databases involved in the research. C1QC expression in pan-cancer and para-carcinoma tissues was studied with the use of TIMER (Li et al., 2020) and TCGA portal (Xu et al., 2019). Based on the immunohistochemical data of normal and KIRC patients, C1QC expression was studied using the Human Protein Atlas database (Karlsson et al., 2021) that comprises transcriptome and proteome data in accordance with investigation of immunohistochemistry and RNA sequencing. For the comparison of the C1QC expression in KIRC patients of different sample types, gender, race, age, tumor stages, tumor grades, subtypes, and lymph node metastatic status, relevant clinical characteristic data and transcriptional expression of C1QC were analyzed by UALCAN (Chandrashekar et al., 2022). The Wilcoxon rank sum test assisted in the assessment of the difference significance.

**TABLE 1:** Summary of databases used in this study.

Name	Link
TIMER	https://cistrome.shinyapps.io/timer/
TCGA portal	http://tumorsurvival.org/
The Human Protein Atlas	https:/www.proteinatlas.org/
UALCAN	http://ualcan.path.uab.edu/
Kaplan-Meier plotter	https://kmplot.com/analysis/
Metascape	http://metascape.org/gp/index.html#/main/step1
STRING	https:/cn.string-db.org/
TISCH	http://tisch.comp-genomics.org/
TISIDB	http:/cis.hku.hk/TISIDB/index.php

### Survival analysis

Kaplan-Meier plotter (Lánczky and Győrffy, 2021) is a reliable tool for the evaluation of genes and survival parameters in tumors. In this study, associations of C1QC expression with overall survival (OS) and relapse-free survival (RFS) were analyzed by Kaplan-Meier plotter. The comparison was drawn on the two patient groups with the use of the Kaplan-Meier survival plot, and the result can be conducive to determining the hazard ratios (HR) with 95% confidence intervals (CI) and log rank *p*-values. Further analysis was performed on the association of C1QC expression with tumor grade in the UALCAN database.

### C1QC interaction and functional enrichment analysis

Metascape (Zhou et al., 2019) refers to a web portal that provides protein interaction network structure analysis, pathway enrichment analysis, and rich gene annotation functions using data from more than 40 bioinformatics knowledgebases. STRING (Szklarczyk et al., 2021) is probably the most comprehensive protein-protein interaction (PPI) data source, covering physical interactions and genetic interactions. The above interactions are calculated according to computational predictions, organism knowledge transfer, as well as interactions adapted from other (primary) databases. Metascape and STRING served for the generation of an interaction network regarding C1QC with other essential proteins and pathways. UALCAN again assisted in exploring the association of C1QC with other genes in KIRC.

### Immune-related analysis of C1QC

TISCH (Sun et al., 2021) acts as an RNA-sequencing database that ensures the investigation on TME across different cancer types based on the particular cell-type annotation. We used TISCH to explore C1QC expression in different cell types across different KIRC datasets. TIMER serves as a platform for comprehensive analysis of tumor-infiltrating immune cells, such as B cells, CD4 + T cells, CD8 + T cells, macrophages, neutrophils, and dendritic cells. We estimated the association of C1QC expression with immune infiltrations in KIRC by exploring the TIMER database. TISIDB (Ru et al., 2019) is a web-based database combining different heterogeneous data types specific to the tumor and immune system interaction, which, in the study, served for assessing the Spearman correlation between C1QC expression and immunoinhibitors, immunostimulators, and chemokines.

### Cell culturing and transfecting processes

The KIRC cell lines 786-O and ACHN presented in this study were obtained from the National Collection of Authenticated Cell Cultures (Shanghai, China). 786-O and ACHN cells underwent culturing treatment in RPMI 1640 medium that involved 10% FBS under 37°C in a 5% CO_2_ chamber that covered 100 mg/mL streptomycin and 100 IU/mL penicillin. SiRNA (si-NC) and small interfering RNA against C1QC (si-C1QC) came from GeneChem. The Opti-MEM and the RNAi Fectin™ solution served for cell transfection. The obtained cells underwent 2 days of post-transfection.

### Quantitative real-time polymerase chain reaction (qRT-PCR)

Trizol reagent (Invitrogen, Grand Island, NY) was adopted to extract total RNA from si-C1QC-transfected cells and si-NC-transfected cells. In accordance with the instruction of the manufacturer, the PrimeScript One Step RT reagent Kit (Takara, RR064A) was adopted to synthesize the cDNA. SYBR Green Real-Time PCR Master Mix (Toyobo, QPK201) and a StepOnePlus Real-Time PCR System (Applied Biosystems) were adopted to perform qRT-PCR. The thermocycling sequence conditions included 95°C for 30 s, followed by 45 cycles of 95°C for 10 s as well as 55°C for 1 min. Glyceraldehyde 3-phosphate dehydrogenase (GAPDH) expression was adopted to normalize all results. The 2^−ΔΔCt^ method was adopted to determine the fold change in comparison with the mean value.

### Western blotting assay

RIPA Lysis Buffer (Biosharp, Beijing, China) was adopted to lyse the above transfected cells. A 10% SDS-PAGE gel was adopted to separate the proteins through electrophoretic process. The separated product was placed into a PVDF membrane (Biosharp, Beijing, China). The respective secondary antibodies (Sangon Biotech, Shanghai, China) and the respective primary antibody (e.g., anti-C1QC and anti-GAPDH) (Proteintech, Wuhan, China; 1:2000 dilution) was adopted to achieve the 10-h incubation of the membranes after 5% evaporated skimmed milk was used to block the membranes. A high sensitivity plus ECL luminescence reagent (Sangon Biotech, Shanghai, China) was employed to detect the target bands after Tris-buffered saline Tween (TBST) was adopted to wash the membranes 3 times.

### Transwell migration and invasion assays

In accordance with the guideline of producer, we seeded transfected 786-O and ACHN cells in 200 μL of RPMI 1640 medium free of serum. The transwell chamber was paved using Matrigel mix for invasion test instead of for migration test. RPMI 1640 medium and 10% FBS served as a KIRC cells chemoattractant after being introduced into the bottom chamber. After 1 day of incubation, the upper chambers underwent fixation, followed by 15 min of crystal violet staining. The images of the cell lines were taken, and they were counted within three fields for visualization.

### Wound healing assay

786-O and ACHN cells were first seeded on six-well culture plate, and then underwent transfection. A standard 20 μL pipette tip served for eliminating the artificial linear wounds on the fused cell monolayer. That was followed by the gradual removal of free-floating cells and debris from the well bottom. After the medium was introduced, the plate underwent incubation at 37°C. One inverted microscope was employed to record the scratch gap area, and we photographed the recorded area at 0 and 48 h. The experiment was performed three times independently for distinguishing the quantitative cell migrating area and the original wound area.

### Cell proliferation experiments

In the Cell Counting Kit-8 (CCK-8) test, 786-O and ACHN cells were first transfected and incubated at 37°C. Subsequently, we introduced CCK-8 solution into the introduction within each well. The well was incubated 2 h. The examination of absorbance was performed at 0, 24, 48, and 72 h at 450 nm.

### Statistical analysis

One individual *t*-testing process served for comparatively analyzing the continuing information of the two groups. GraphPad Prism 8.0 served for the statistical analysis. *p*-value <0.05 reported statistical significance.

## Results

### C1QC is highly expressed in KIRC

**FIGURE 1:**
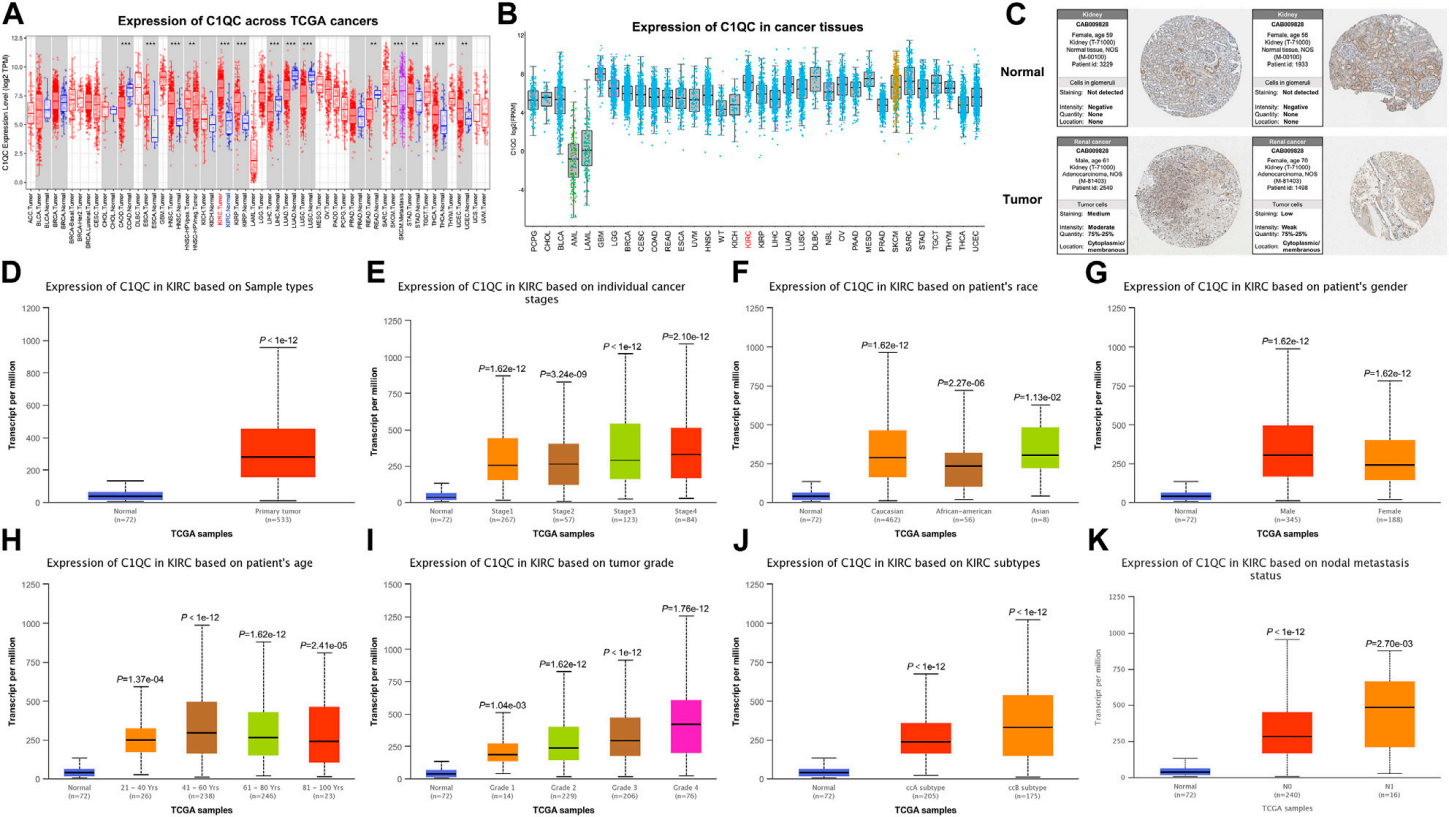
C1QC expression overview. **(A)** C1QC mRNA expression in a variety of cancer tissues in comparison with normal tissues (^*^ *p* < 0.05, ^**^ *p* < 0.01, ^***^ *p* < 0.001). **(B)** C1QC mRNA expression in a variety of cancers. **(C)** C1QC protein expression in normal kidney tissues and KIRC tissues. **(D–K)** C1QC mRNA expression difference based on sample types, tumor stages, race, gender, age, tumor grades, subtypes, and lymph node metastatic status. The Wilcoxon rank sum test served for the assessment of the difference significance.

We initially studied the changes of C1QC expression levels between different tumor and adjacent normal tissues using the RNA-seq data from TCGA database. The analysis demonstrated that the expression levels of C1QC in READ (Rectum Adenocarcinoma), LUSC(Lung Squamous Cell Carcinoma), LUAD (Lung Adenocarcinoma), LIHC (Liver Hepatocellular Carcinoma), and COAD (Colon Adenocarcinoma) decreased notably, whereas those in KIRC, UCEC (Uterine Corpus Endometrial Carcinoma), THCA (Thyroid Carcinoma), STAD (Stomach Adenocarcinoma), KIRP (Kidney Renal Papillary Cell Carcinoma), HNSC (Head and Neck Cancer), and ESCA (Esophageal Carcinoma) increased significantly (Figure 1A). Then, further investigation was conducted on C1QC expression in a diverse set of cancer tissues. KIRC presented higher C1QC expression in comparison with most other cancers (Figure 1B). According to data from Human Protein Atlas database, there were different levels of protein expression intensity of C1QC in kidney cancer tissues but not in normal kidney tissues (Figure 1C). Furthermore, subgroup analysis based on different sample types, tumor stages, race, gender, age, tumor grades, subtypes, and lymph node metastatic status indicated that KIRC patients had obviously higher C1QC mRNA level in comparison with healthy individuals (Figures 1D–K). It is noteworthy that C1QC expression showed an upward trend with higher tumor grade.

### Prognostic significance of C1QC in KIRC

**FIGURE 2:**
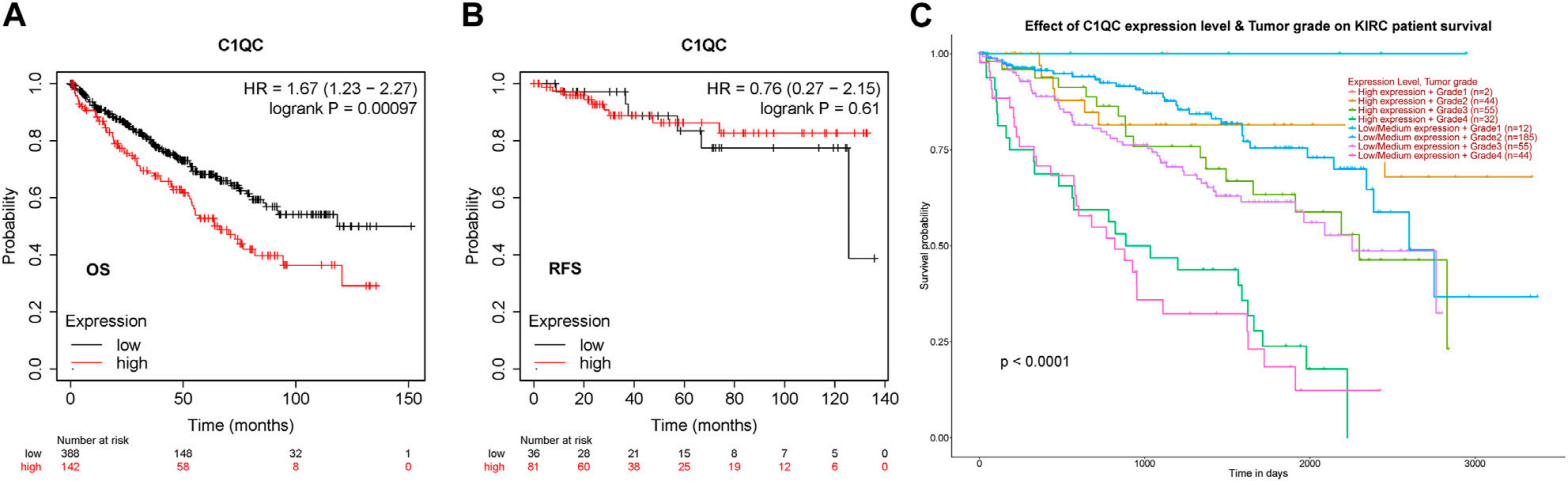
Clinical significance of C1QC in KIRC. **(A, B)** The association of C1QC expression with KIRC patients’ OS and RFS. **(C)** The association of C1QC expression with KIRC tumor grades.

The prognostic significance of C1QC in KIRC was investigated using Kaplan-Meier plotter. As found, high level of C1QC reported shorter OS (HR = 1.67 (1.23–2.27), logrank *p* = 0.00097), but no significant association with RFS (HR = 0.76 (0.27–2.15), logrank *p* = 0.61) was detected (Figures 2A, B). TCGA database indicated that C1QC expression and tumor grade had synergistic effect on KIRC patients’ prognosis (Figure 2C), that conformed to the data in Figure 1I. Accordingly, C1QC serves as a hazard for the prediction of KIRC patients’ poor prognosis.

### C1QC promotes KIRC cells *in vitro*

**FIGURE 3:**
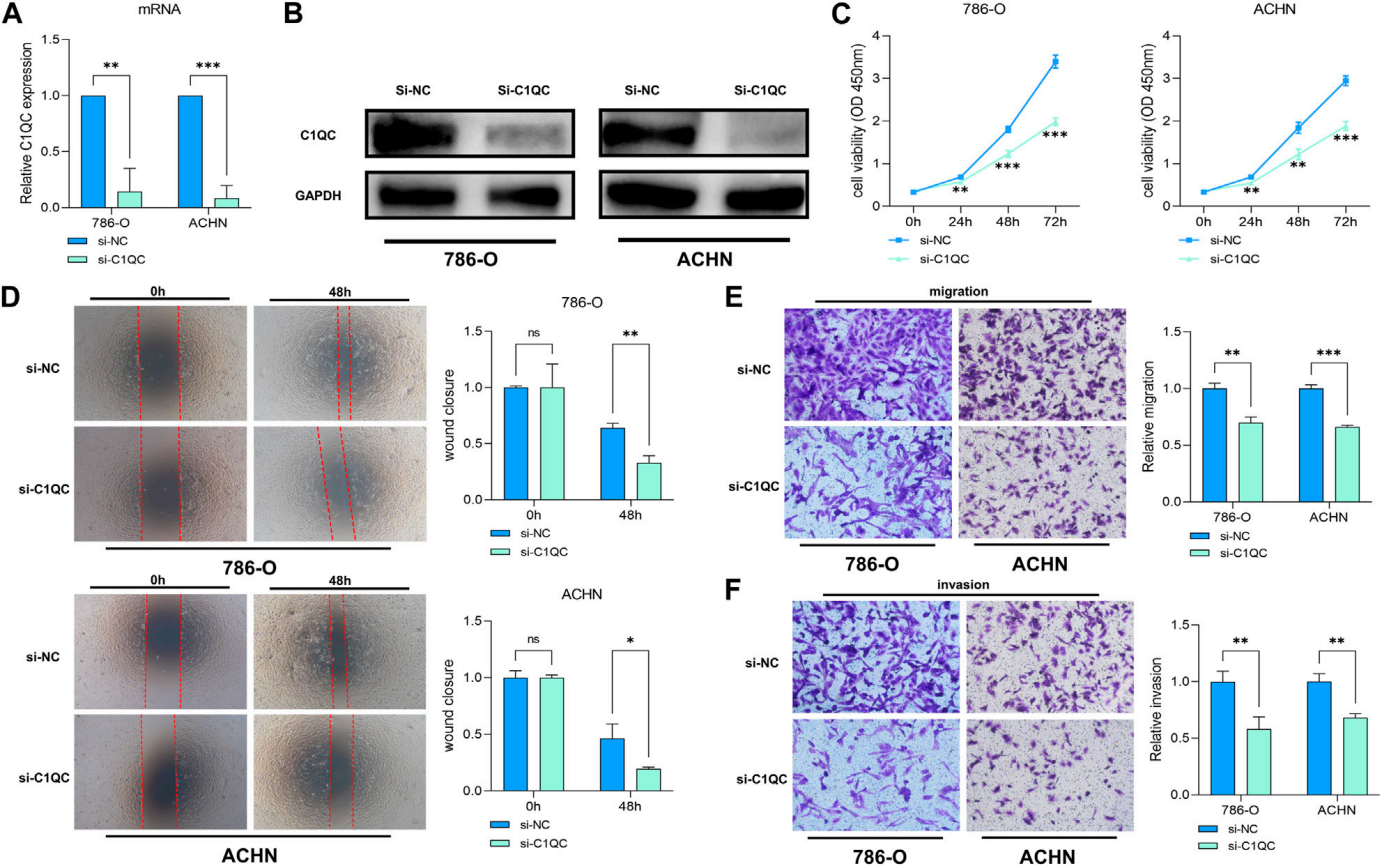
C1QC knockdown inhibits KIRC cell development. **(A, B)** The efficiency of C1QC knockdown is evaluated by qRT-PCR and Western blotting assay. **(C)** C1QC knockdown inhibits KIRC cell proliferation. **(D)** Wound healing assay of C1QC knockdown. **(E)** C1QC knockdown inhibits KIRC cell migration. **(F)** C1QC knockdown inhibits KIRC cell invasion. (^∗^ *p* < 0.05, ^∗∗^ *p* < 0.01, ^∗∗∗^ *p* < 0.001).

To confirm the oncogenic activity of C1QC we identified through bioinformatics analyses in KIRC, the widely used KIRC cell lines, 786-O and ACHN with stable C1QC knockdown were successfully constructed. Knockdown efficiency of C1QC was confirmed through qRT-PCR (Figure 3A) and Western blotting assay (Figure 3B). CCK-8 (Figure 3C) assay showed that C1QC knockdown significantly inhibited the proliferation of 786-O and ACHN cells compared with the control group. According to wound healing assay, in the KIRC cell lines, C1QC inhibition presented an obviously lower wound closure rate in comparison with the control group (Figure 3D). In comparison with the control group in the confluence monolayer transwell experiment regarding cultured KIRC cell lines, si-C1QC could suppress the relative migration and invasion rate (Figures 3E, F). As revealed by the above results, C1QC knockdown is capable of inhibiting KIRC proliferation, migration, and invasion *in vitro*.

### Genes and proteins co-interacted with C1QC are relevant to signaling pathways affecting immune system

**FIGURE 4:**
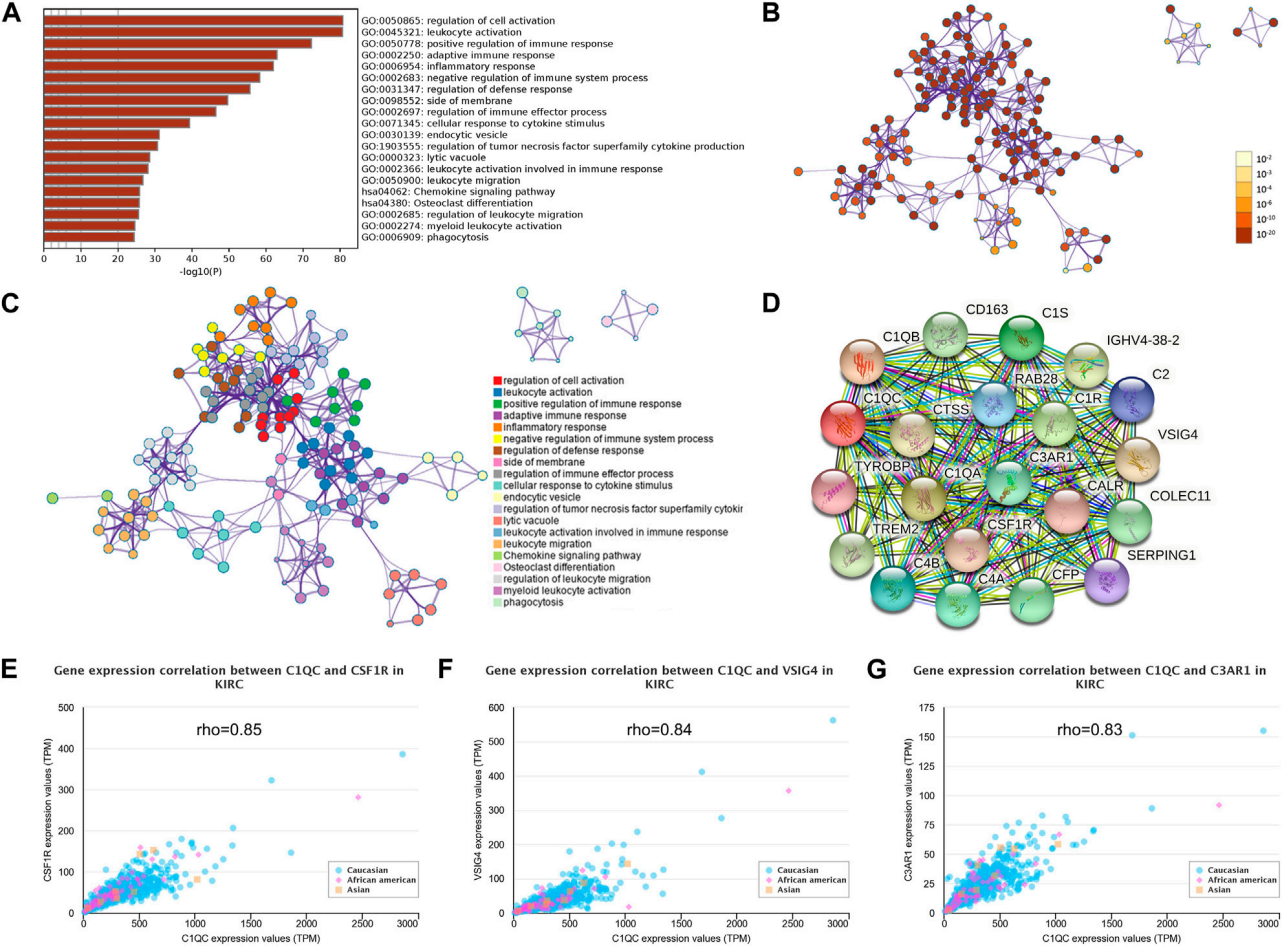
Genes and proteins co-interacted with C1QC show a relevance to immune-related signaling pathway. **(A)** KEGG and GO analysis results of genes co-expressed with C1QC in KIRC. **(B)** *p*-value colored network of enriched terms; *p* values for terms that have a larger number of genes are usually smaller. **(C)** Cluster ID-colored network of enriched terms; nodes sharing the same cluster ID usually approach to each other. **(D)** C1QC-proteins interaction. **(E–G)** Association analysis of C1QC with CSF1R, VSIG4, and C3AR1 in different ethnic groups.

Pathways enrichment analysis on co-expression genes were accomplished by GO and KEGG (Metascape). The functional pathways enrichment heatmap revealed that C1QC was closely linked with immune-related signaling pathways such as regulation of cell activation (GO:0050865), leukocyte activation (GO:0045321), positive regulation of immune response (GO:0050778), adaptive immune response (GO:0002250), inflammatory response (GO:0006954), negative regulation of immune system process (GO:0002683), regulation of defense response (GO:0031347), regulation of immune effector process (GO:0002697), cellular response to cytokine stimulus (GO:0071345), regulation of tumor necrosis factor superfamily cytokine production (GO:1903555), leukocyte activation involved in immune response (GO:0002366), leukocyte migration (GO:0050900), Chemokine signaling pathway (hsa04062), and phagocytosis (GO:0006909) (Figures 4A–C). A STRING interactive network was built to recognize proteins capable of interacting with C1QC (Figure 4D). Next, further study displayed that C1QC expression was obviously relevant to the proteins possibly interacting with C1QC (CSF1R, VSIG4, and C3AR1) (Figures 4E–G).

### C1QC expression at single-cell level

**FIGURE 5:**
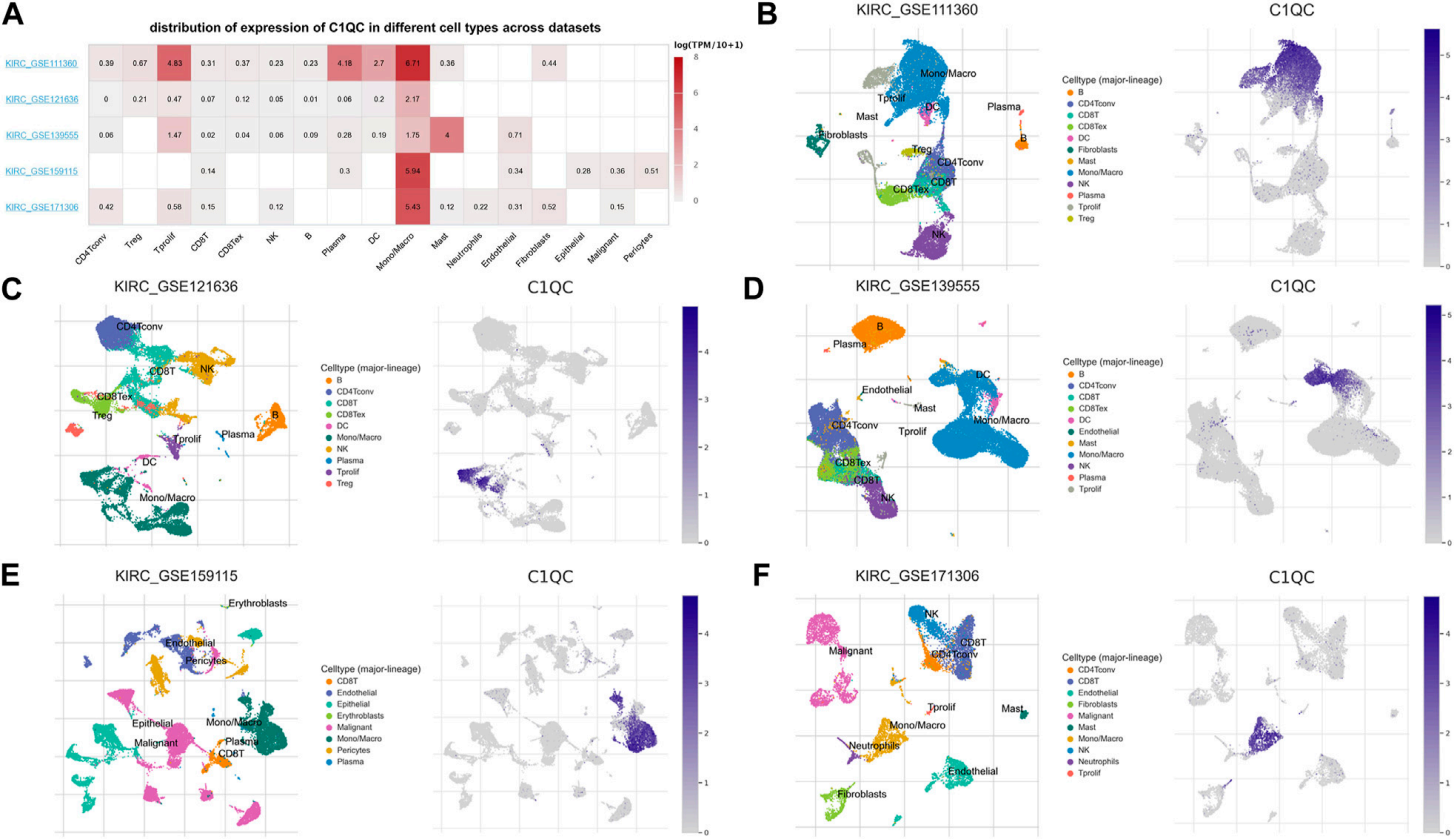
C1QC at single-cell level. **(A)** Heatmap demonstrates C1QC expression in cells from a variety of databases. **(B–F)** C1QC single-cell cluster in different databases.

The study primarily investigated the C1QC expression at the single-cell level. The expression of C1QC in TME-related immune cells was analyzed using the TISCH database’s five datasets (KIRC-GSE111360, KIRC-GSE121636, KIRC-GSE139555, KIRC-GSE159115, and KIRC-GSE171306). In distribution heatmap (Figure 5A), we found low to moderate C1QC expression in immune cells (e.g., neutrophils, B cells, natural killer T cells, CD8^+^ T cells, CD4^+^ T cells, Tregs, and dendritic cells). C1QC was primarily expressed at the macrophages cluster except in KIRC-GSE139555 (in which mast cells showed the highest expression). We then analyzed the above datasets using single-cell cluster map, which were divided into various types of cells. As depicted in Figures 5B–F, C1QC expression level remained the highest in macrophages, consistent with the results shown in Figure 5A. Accordingly, C1QC expression level was quite different in distinct cell types with the highest in macrophages instead of KIRC cells, suggesting that C1QC may also play its role in immune cells besides cancer cells.

### C1QC expression is relevant to immune infiltration in KIRC

**FIGURE 6:**
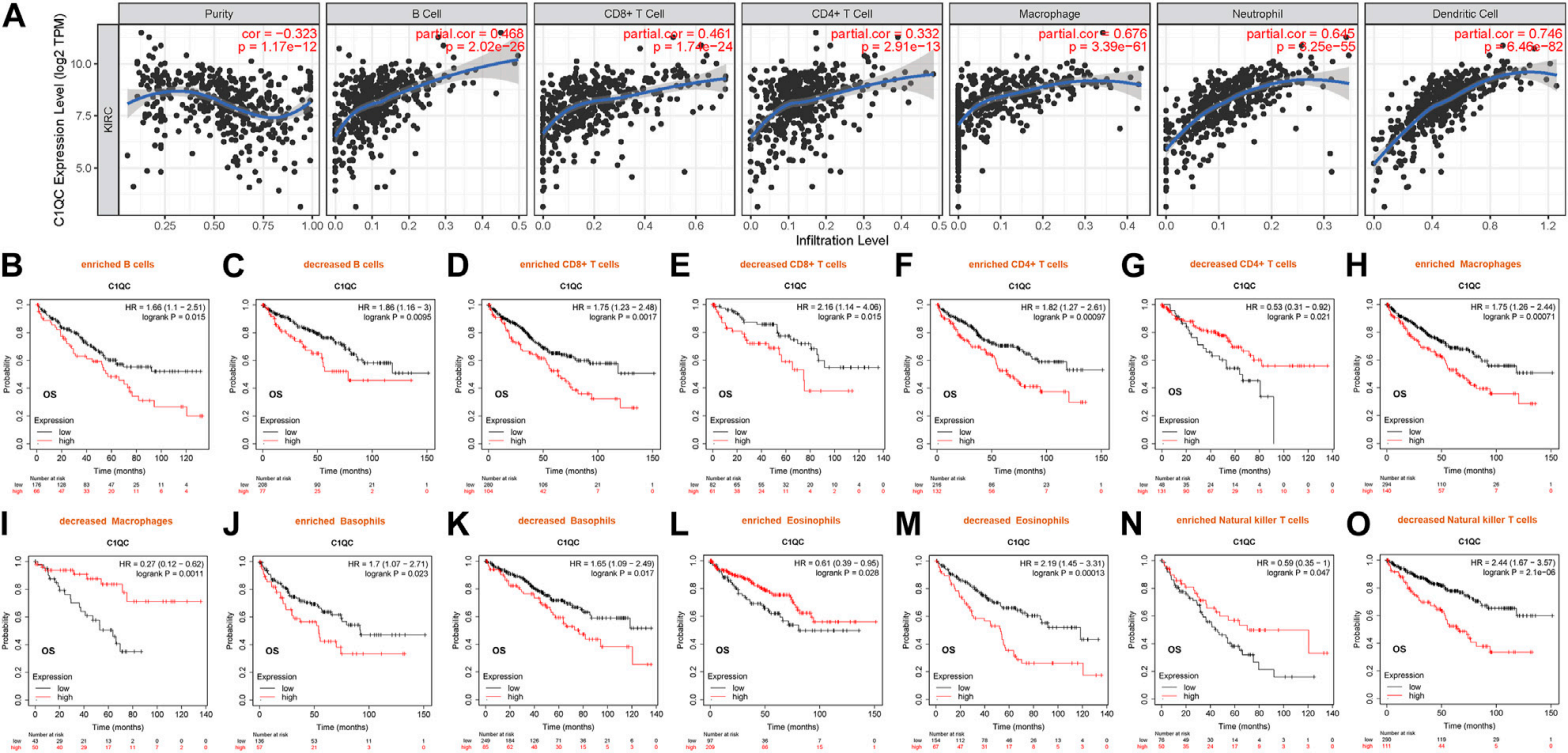
Associations of C1QC expression with immune infiltration and KIRC prognosis. **(A)** Levels of immune infiltration of B cell, CD8^+^ T cell, CD4^+^ T cell, macrophage, neutrophil, and dendritic cell in KIRC. **(B–O)** Associations of C1QC expression of different immune cells subgroups with prognoses in KIRC.

Existing research has suggested that immune infiltration can affect renal cancer prognosis (Zhang et al., 2019). Therefore, the Spearman correlation served for analyzing the association of C1QC transcription level and the measured immune cell infiltration level in KIRC. As found, C1QC expression presented a negative relevance to the purity of KIRC (rho = −0.323, *p* = 1.17e^−12^). However, high C1QC expression showed a strong relevance to the infiltrating degree of B cell (rho = 0.468), CD8^+^ T cell (rho = 0.461), CD4^+^ T cell (rho = 0.332), macrophage (rho = 0.676), neutrophil (rho = 0.645), and dendritic cell (rho = 0.746) (Figure 6A). Notably, they had *p* values far less than 0.001. Accordingly, C1QC can serve as an important tumor immune infiltration regulator in KIRC.

### Prognostic significance of C1QC in KIRC based on immune cells

This study has identified the relevance of C1QC expression to the immune infiltration of KIRC. Also, C1QC up-expression reported worse KIRC prognosis. Hence, C1QC might affect KIRC patients’ prognosis partly because of immune infiltration. We did prognosis analyses of C1QC expression in KIRC considering immune cells subgroups *via* Kaplan-Meier plotter again, finding that high C1QC expression of KIRC in enriched (HR = 1.66) or decreased B cells (HR = 1.86), enriched (HR = 1.75) or decreased (HR = 2.16) CD8^+^ T cells, enriched CD4^+^ T cells (HR = 1.82), enriched macrophages (HR = 1.75), enriched (HR = 1.7) or decreased (HR = 1.65) basophils, decreased eosinophils (HR = 2.19), and decreased natural killer T cells (HR = 2.44) cohorts had a worse prognosis respectively (Figures 6B–F, H, J, K, M, O). Contrarily, high C1QC level in KIRC had favorable prognosis in decreased CD4^+^ T cells (HR = 0.53), decreased macrophages (HR = 0.27), enriched eosinophils (HR = 0.61), and enriched natural killer T cells (HR = 0.59) subgroups respectively (Figures 6G, I, L, N). Of note, high C1QC expression induced an opposite effect on OS of enriched CD4^+^ T cells and decreased CD4^+^ T cells subgroups. This similar effect of C1QC expression was also observed in KIRC patients categorized based on macrophages, eosinophils, and natural killer T cells subgroups respectively. On that basis, high C1QC expressions in KIRC may affect prognoses partly through immune infiltration.

### C1QC expression is associated with immune factors

**FIGURE 7:**
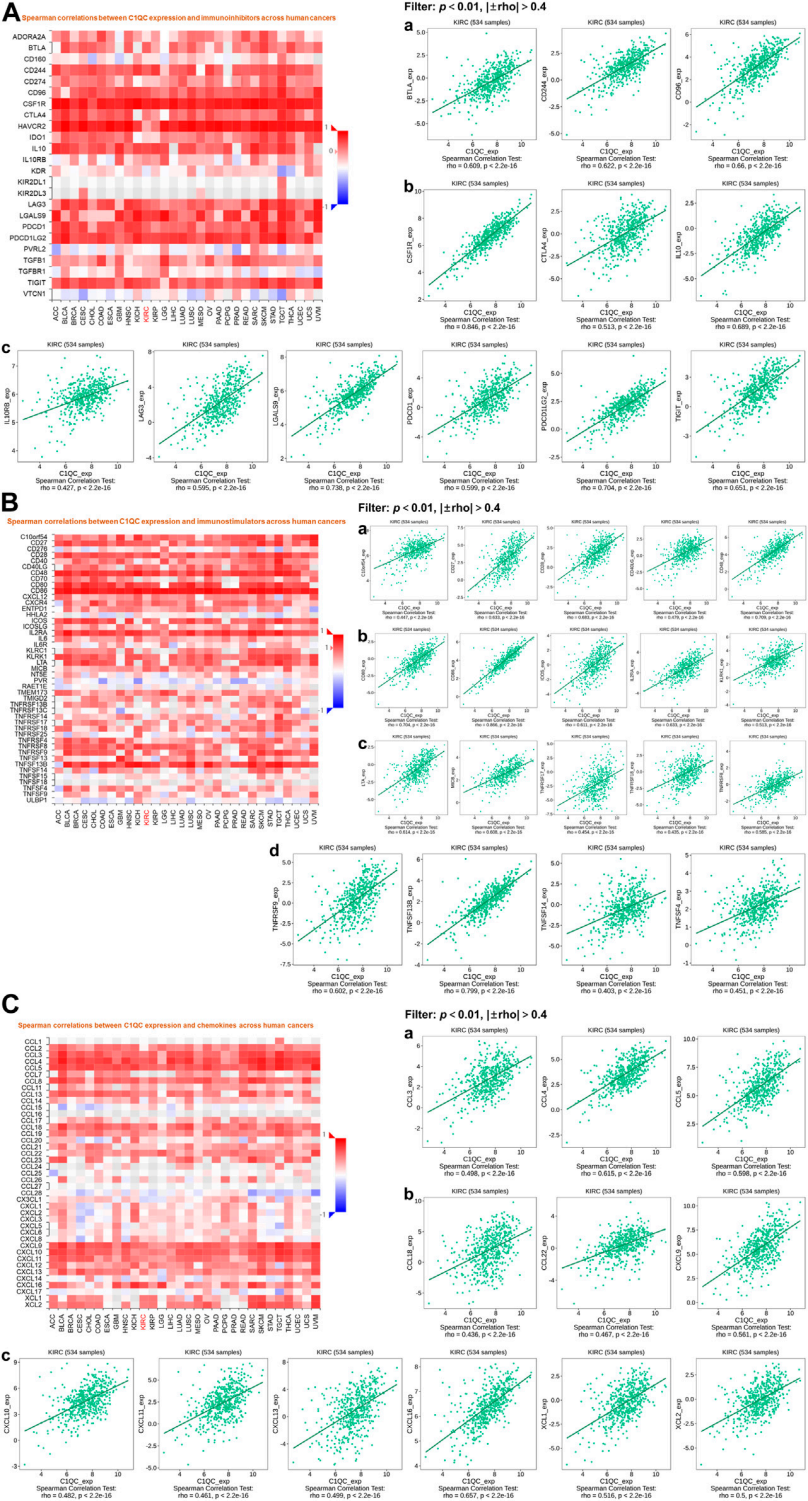
Associations of C1QC expression with 3 cancer-related immune factor types in KIRC. **(A)** Immunoinhibitors. **(B)** Immunostimulators. **(C)** Chemokines. (**A–C**: Heat maps show associations of C1QC and immune factors in different cancers; **a–d**: Line graphs show associations of C1QC with specific immunomodulators in KIRC).

More and more evidences have shown the crucial role of the immune system in cancer process (Candeias and Gaipl, 2016), which conforms to our finding from the pathways enrichment analysis for C1QC in Metascape. Therefore, we further investigated associations of C1QC expression and immune factors. After the filtering taking *p* < 0.01 and |±rho| > 0.4 as criteria, the immune factors including immunoinhibitors (BTLA, PDCD1 (PD1), CTLA4, etc.), immunostimulators (C10orf54, CD27, CD28, etc.), and chemokines (CCL3, CCL4, CCL5, etc.) which were strongly associated with C1QC expression of KIRC are shown in Figure 7.

## Discussion

KIRC is a representative subtype of RCC, threatening the health of hundreds of thousands of people globally each year (Bukavina et al., 2022). KIRC often exhibits a poor prognosis for the high resistance to chemotherapy and radiotherapy (Cohen and McGovern, 2005). Understanding the molecular mechanisms underlying KIRC pathogenesis may assist the discovery of valuable diagnostic and prognostic biomarkers and the development of effective therapeutic targets. The complement system connects innate and adaptive immunity, which plays a critical role in maintaining homeostasis. Although complement activation is capable of resisting pathogen invasion and protecting the host, complement also plays a tumor-promoting function (Roumenina et al., 2019). C1QC, as a subunit of C1 which is the first complement structure, promotes cancer progression, as demonstrated previously. In colon carcinoma, C1QC regulates immune infiltration of macrophages, and then affects neutrophil activation, resulting in tumor progression (Deng et al., 2022). Also, a high proportion of C1QC-expressing tumor-associated macrophages (TAM) in the TME of colon cancer suggests a poor clinical outcome (Liu Y. et al., 2022). In lung squamous cell carcinoma, C1QC induced CD8^+^ T cell exhaustion by up-regulating the immunosuppressive TOX pathway genes, reducing OS (Zhang et al., 2021). Notably, C1QC protein expression presented a considerable increase in KIRC and a positive relevance to an advanced stage of disease (Zhang et al., 2016). This study verified the oncogenetic role of C1QC in KIRC. However, the specific role of C1QC in KIRC patients has been rarely investigated. Bioinformatics provide researchers with large and complex biological data, which guides gene exploration and assists clinical diagnosis, treatment, and prognosis prediction. The role played by C1QC expression in prognosis and tumor immunity of KIRC patients was explored in depth through bioinformatic analyses using public databases and the validation of basic experiments.

In our present study, we employed TIMER and TCGA portal databases to perform pan-cancer analysis on the transcription levels of C1QC, finding the obvious upregulation of C1QC expression in KIRC, in comparison with normal tissues. We then verified differential protein expression using Human Protein Atlas database and reached a consistent conclusion. Furthermore, we investigated associations of C1QC expression and clinicopathological characteristics in KIRC patients. KIRC patients presented considerably higher C1QC mRNA levels in comparison with normal tissues in accordance with the subgroup analysis based on different sample types, tumor stages, race, gender, age, tumor grades, subtypes, and lymph node metastatic status. Kaplan-Meier plotter database was applied to a survival analysis for confirming whether C1QC can predict the prognosis, finding that in KIRC, poorer OS was relevant to higher C1QC expression. Moreover, C1QC expression upregulation in tumor grade could report poor KIRC prognosis. On that basis, C1QC can predict the KIRC prognosis biologically.

To further examine the mechanism of C1QC in promoting KIRC development, functional annotations and PPI network were constructed. Results showed that C1QC protein, which may interact with CSF1R, VSIG4, and C3AR1, generally mapped to immune-related activities through GO and KEGG enrichment analyses. The positive associations of C1QC with CSF1R, VSIG4, and C3AR1 in KIRC were confirmed in our research. In contrast to C3AR1 (Chalbatani et al., 2022; Chu et al., 2020), high expression levels of CSF1R (Yang et al., 2016) and VSIG4 (Hu et al., 2019) both could negatively affect the survival of KIRC patients. Existing research has suggested that high mRNA expression of C1QC and CSF1R is associated with immunosuppression in the lung squamous cell carcinoma tissue microenvironment and worsen patient survival outcomes (Zhang et al., 2021). Notably, patients with anti-PD-1/PD-L1-resistant advanced RCC achieved clinical benefit in a phase I trial of CSF1R inhibitor (cabiralizumab) and CD40 agonist (sotigalimab) (Weiss et al., 2021). Taken together, CSF1R and VSIG4 may exert a synergistic effect on C1QC and lead to the poorer prognosis of KIRC patients through immune-related pathways.

Emerging studies have evidenced that immune system is involved in the thrive of malignant tumors (Candeias and Gaipl, 2016). The TME is a complex microenvironment formed during the fight between tumor cells and immune system, which is contributory to tumor growth and metastasis, and finally facilitates tumor immune escape. Single-cell RNA-sequencing provides more insights into the cell behavior in the TME, suggesting the evolutionary nature of cancer (Jiang et al., 2019). The single-cell analysis indicated that C1QC had a primary expression in macrophages instead of KIRC cells in tumor samples. This is the first study to explore C1QC expression in different cell clusters specific to cancer research. Fang Hong et al. conducted a single-cell pan-cancer analysis on C1QC and macrophages. They confirmed that the dysfunctional CD8^+^ T cell abundance may be adjusted by a certain subset of TAMs expressing higher C1QC through cytokine-mediated signaling (Hong et al., 2021). Zhou Liu et al. confirmed a certain subset of macrophages, i.e., macrophages associated with lipids, which highly express C1QC and exhibit an immunosuppressive effect and enhanced phagocytosis in the tumor-adipose microenvironment of breast cancer. Lipid-associated macrophages depletion potentiated the anti-tumor effect exerted by anti-PD1 therapy in the allograft cancer mouse models (Liu Z. et al., 2022). The above results indicated an oncogenic role of C1QC in macrophage polarization and revealed the cell type-specific role played by RNA editing.

Besides, the study confirmed the direct relevance of C1QC to immune infiltration degree in KIRC. In the TME, immune cell infiltration critically affects the cancer process. The result of *in vitro* experiments suggested that C1QC expression in proliferation, migration, and invasion of KIRC cells was required. Nevertheless, researches have not confirmed the relevance of C1QC expression to immune infiltration in KIRC. On that account, we focused on investigating such relevance, finding that C1QC expression in KIRC presented a positive association with tumor-infiltrating immune cells like B cell, CD8^+^ T cell, CD4^+^ T cell, macrophage, neutrophil, and dendritic cell. Accordingly, C1QC regulated the infiltration of immune cells into the TME in KIRC tissues. Among the above immune cells, high C1QC expression in KIRC reported a poorer prognosis in enriched CD4^+^ T cells and macrophages subgroups in contrast to a fine prognosis in enriched eosinophils and natural killer T cells cohorts. Additionally, no matter in enriched or decreased B cells, CD8^+^ T cells, and basophils subgroups, high C1QC expression shortened the OS of KIRC patients. CD4 + T cells can enhance KIRC cell proliferation *via* activating YBX1/HIF2α signaling pathway (Wang et al., 2018). TREM2/APOE/C1Q-positive macrophage infiltration potentially indicates the prognosis of KIRC (Obradovic et al., 2021). Eosinophil levels and relative eosinophil change are associated with a good therapeutic effect of nivolumab for metastatic RCC (Herrmann et al., 2021). Furthermore, natural killer T cell is an important component of antitumor effect of secreted IL-21 on RCC (Kumano et al., 2007). According to previous studies, the proportion of CD4^+^ T cells (Lee et al., 2020) and macrophages (Wang et al., 2020) indicates inferior clinical outcome in patients with RCC respectively. The contrary effects of Eosinophils (Herrmann et al., 2021) and natural killer T cells (Geissler et al., 2015) were also confirmed respectively in other studies. The above results may explain that C1QC can shorten the OS of KIRC patients partly by mediating immune cell infiltration.

Finally, we identified a lot of C1QC related immunoinhibitors, like BTLA, PDCD1 (PD1), and CTLA4, which showed a positive relevance to C1QC expression. Meanwhile, some immunostimulators such as C10orf54, CD27, and CD28 and chemokines such as CCL3, CCL4, and CCL5 were also found to exhibit a positive relevance to C1QC expression. The above findings confirmed that C1QC could notably regulate KIRC from immune perspective. Hence, we speculated that combining these immunological checkpoint inhibitors and C1QC inhibitors may effectively enhance the anti-tumor effect for KIRC patients.

The association of C1QC with KIRC was studied, whereas some limitations should be acknowledged. First, this study was primarily based on bioinformatics. Research results may be affected by the constant update and expansion of online platform databases. Second, experiments should be urgently conducted to validate our analysis results. The function of C1QC and its underlying mechanisms in different cell types and cancers should be examined in depth.

## Conclusion

In brief, the study is the first one that, through bioinformatics, demonstrated the strong relevance of the elevated C1QC to clinicopathological features, poor prognosis, and enhanced immune infiltration degree in KIRC. Furthermore, C1QC may affect the KIRC prognosis by virtue of a new mechanism, i.e., tumor immune infiltration, that contributes to a novel perspective for further in-depth research on immunotherapy of KIRC.

## Data Availability

The datasets presented in this study can be found in online repositories. The names of the repositories can be found in the article.
